# Association of Polymorphisms in the Human *IL4* and *IL5* Genes With Atopic Bronchial Asthma and Severity of the Disease

**DOI:** 10.1002/cfg.293

**Published:** 2003-06

**Authors:** Maxim B. Freidin, Olga S. Kobyakova, Ludmila M. Ogorodova, Valery P. Puzyrev

**Affiliations:** 1 Population Genetics Laboratory Research Institute for Medical Genetics 10 Nab. r. Ushaiky Ave. Tomsk 634050 Russia; 2 Department of Pediatrics Siberian State Medical University Tomsk Russia; 3 Department of Medical Genetics Siberian State Medical University Tomsk Russia

## Abstract

Two polymorphisms in the *IL4* (G/C 3′-UTR) and *IL5* (C-703T) genes were studied in a sample of families whose probands had atopic bronchial asthma (BA) (66 families, n = 183) and in a group of non-cognate individuals with the severe form of the disease (n = 34). The samples were collected from the Russian population in the city of Tomsk (Russia). Using the transmission/disequilibrium test (TDT), a significant association of allele C-703 *IL5* with BA was established (TDT = 4.923, *p* = 0.007 ± 0.0007). The analysis of 40 individuals with mild asthma and 49 patients with the severe form of the disease revealed a negative association of genotype GG *IL4* (OR = 0.39, 95% CI = 0.15−0.99, *p* = 0.035), and also a trend towards a positive association of the GC *IL4* genotype (OR = 2.52, 95% CI = 0.98−6.57, *p* = 0.052) with mild BA. There was a concordance of the clinical classification of BA severity with the ‘genotype’ (McNemar’s χ^2^ test with continuity correction constituted 0.03, d.f. = 1, *p* = 0.859). These results suggest that polymorphisms in the *IL4* and *IL5* genes contribute to the susceptibility to atopic BA and could determine the clinical course of the disease.


**Comparative and Functional Genomics**
*Comp Funct Genom* 2003; **4**: 346–350.
Published online in Wiley InterScience (www.interscience.wiley.com). **DOI:** 10.1002/cfg.293

**Short Communication**

### Association of polymorphisms in the human *IL4* and *IL5* genes with atopic bronchial asthma and severity of the disease

Maxim B. Freidin[1], Olga S. Kobyakova[2], Ludmila M. Ogorodova[2] and Valery P. Puzyrev[1,3]*

[1] *Population Genetics Laboratory, Research Institute for Medical Genetics, Tomsk, Russia*
[2] *Department of Pediatrics, Siberian State Medical University, Tomsk, Russia*
[3] *Department of Medical Genetics, Siberian State Medical University, Tomsk, Russia*

**Correspondence to: Valery P. Puzyrev, Research Institute for Medical Genetics, 10 Nab. r. Ushaiky Ave., 634050 Tomsk, Russia. E-mail: valery@img.tsu.ru.*

### Abstract

**Two polymorphisms in the *IL4* (G/C 3′-UTR) and *IL5* (C-703T) genes were studied in a sample of families whose probands had atopic bronchial asthma (BA) (66 families, $n = 183$) and in a group of non-cognate individuals with the severe form of the disease ($n = 34$). The samples were collected from the Russian population in the city of Tomsk (Russia). Using the transmission/disequilibrium test (TDT), a significant association of allele C-703 *IL5* with BA was established (TDT = 4.923, $p = 0.007 \pm 0.0007$). The analysis of 40 individuals with mild asthma and 49 patients with the severe form of the disease revealed a negative association of genotype GG *IL4* (OR = 0.39, 95% CI = 0.15–0.99, $p = 0.035$), and also a trend towards a positive association of the GC *IL4* genotype (OR = 2.52, 95% CI = 0.98–6.57, $p = 0.052$) with mild BA. There was a concordance of the clinical classification of BA severity with the 'genotype' (McNemar's $X^2$ test with continuity correction constituted 0.03, d.f. = 1, $p = 0.859$). These results suggest that polymorphisms in the *IL4* and *IL5* genes contribute to the susceptibility to atopic BA and could determine the clinical course of the disease. Copyright © 2003 John Wiley & Sons, Ltd.**

Accepted: 31 March 2003

**Keywords: atopic bronchial asthma; candidate genes; *IL4*; *IL5*; polymorphism; association**

### Introduction

Atopic bronchial asthma (BA) is a widespread allergy-mediated inflammatory disease of the respiratory tract. From a genetic point of view it is a complex disease whose aetiology and pathogenesis are determined by the interaction of numerous hereditary and environmental factors. Multiple studies carried out during the last 10–15 years have resulted in significant progress in the understanding of the molecular–genetic basis for the predisposition to the disease. Several successful genome-wide screenings have been carried out. About 10 chromosomal loci with significant linkage to the asthma phenotype have been repeatedly reported. Polymorphisms of about 50 candidate genes have been investigated. Experiments on cell lines and model organisms have been conducted (Cookson and Moffatt, 2000). In comparison with other complex diseases, there is probably a better coincidence for BA in genetic data obtained by different scientific groups.

However, genetic susceptibility to BA is still far from being completely understood. Partially, this is because many candidate genes that could potentially contribute to the susceptibility to the disease have not been investigated to date, and not all of the polymorphisms of the candidate genes have been tested for a possible association with the disorder. These circumstances make it necessary to examine new polymorphisms of BA candidate

Copyright © 2003 John Wiley & Sons, Ltd.

genes regarding their contribution to the disease and to its clinical manifestation.

The human *IL4* and *IL5* genes, encoding interleukin-4 and interleukin-5, respectively, are strong candidates for BA because these cytokines are the key molecules for such pathogenetic components of the disease as allergy and eosinophilic inflammation. Both genes are located on chromosome 5q31–33, a region well-known to be in significant linkage with BA and disease associated traits such as serum IgE levels and bronchial hyperresponsiveness. Single nucleotide polymorphisms in the *IL4* and *IL5* genes have recently been described: a G to C transversion in the 3′-untranslated region (3′-UTR) of the *IL4* gene at nucleotide 9291 was found at the Experimental Medicine Unit (headed by Professor Julian M. Hopkin) of the University of Wales, Swansea, UK (T. Shirakawa, personal communication), and a C to T transition at nucleotide 703 upstream of the first ATG codon of the *IL5* gene (C-703T) was found in the genetic analysis of familial eosinophilia (Rioux *et al.*, 1997). Both polymorphisms are located outside of the known control regions of the genes. As far as we are aware, these polymorphisms have not yet been tested for association with BA.

In the present study, an association analysis of BA, and its severity, with the G/C 3′-UTR *IL4* and the C-703T *IL5* polymorphisms has been carried out in a sample of Russian inhabitants of Tomsk, Russia.

### Materials and methods

### Patients

In total, 66 families ($n = 183$) with a child affected by BA were studied. They were interviewed and consent to participate in the study was obtained. The age range of the probands (children with BA) was 1.5–15 years (mean age ± SD constituted $8.4 \pm 3.4$ years). The majority of probands were boys ($n = 46$). The results of physical examination and medical history were collected from 51 trios (two parents and an affected child) and 15 incomplete families (one parent was absent). WHO criteria were used to define the bronchial asthma phenotype in all affected subjects examined in this study (National Heart, Lung and Blood Institute, 1995). Additionally, 34 unrelated patients (16 males, 18 females, mean age ± SD constituted $39.4 \pm 4.4$ years) with a diagnosis of severe atopic BA, based on the same clinical criteria, were included in the study. All individuals were of Russian ethnicity and lived permanently in Tomsk.

### Genotyping

Genotypes were determined by restriction analyses of PCR products using techniques developed in the Experimental Medicine Unit at the University of Wales Swansea. For genotyping of the G/C 3′-UTR polymorphism in the *IL4* gene the following oligonucleotide primers were used: 5′-ctc-agt-aca-cca-tat-ggc-t (nucleotides 9028–9046, according to the published sequence of the human *IL4* gene; GenBank ID, M23442) and 5′-cca-gtg-act-atc-att-ata-att-cc (nucleotides 9628–9606). The PCR mixture contained 2.5 pmol specified primers, 2 mmol each dNTP, 1.2 µl 10× reaction buffer, 1.5 mM $MgCl_2$, 0.5 U *Taq* DNA-polymerase ('SibEnzyme', Russia), and 100–200 ng genomic DNA. PCR conditions were as follows: initial denaturation at 94 °C for 5 min, followed by 33 cycles of annealing at 60 °C (1 min); elongation at 72 °C (45 s); denaturation at 94 °C (45 s); and a final elongation at 72 °C for 8 min. PCR-products were cleaved overnight with 5 U *VneI* (isoschizomer of *ApaLI*; 'SibEnzyme', Russia), and the resulting fragments were resolved on a 2% agarose gel stained with ethidium bromide and visualized under UV light. Homozygotes for allele G were recognized by the presence of two bands of 332 bp and 269 bp, homozygotes for allele C had a single 601 bp band, and heterozygotes had all three bands.

For the genotyping of the C-703T transition in the *IL5* gene the following oligonucleotide primers were used: 5′-cag-gga-gag-cca-atc-agt (nucleotides −857 to −840 according to the published sequence of the human *IL5* gene; GenBank ID, J03478) and 5′-atg-atg-tcc-aga-ctc-c<u>a</u>g-gat-ct (nucleotides −680 to −702; the underlined base is exchanged to incorporate a restriction site for *AlwNI* in the case of allele C). The PCR mixture and the program for amplification were similar to those for G/C 3′-UTR *IL4*. The PCR products were cleaved overnight with 0.013 U *AlwNI* (New England Biolabs, USA). The products of hydrolysis were resolved on a 3% agarose gel stained with ethidium bromide and visualized under UV light. Homozygotes for allele C were recognized by the presence of two bands

Copyright © 2003 John Wiley & Sons, Ltd. 

of 160 bp and 18 bp, homozygotes for allele T had a single 178 bp band, and heterozygotes had all three bands.

### Association analysis

The analysis of association of genetic polymorphisms with BA was carried out by TDT (Spielman *et al.*, 1993). In this test, the numbers of allele transmissions from heterozygous parents to their affected offspring are used to estimate degrees of association. For the evaluation of the significance level for TDT, a Monte Carlo-based procedure was applied, with a 15 000 pseudosample simulation, using the program Nx23 by Yuri Aulchenko (Institute of Cytology and Genetics, Novosibirsk, Russia).

### Results and discussion

In this study, a total of 114 cases of *IL4* transmission and 113 cases of *IL5* transmission were accounted for out of 117 possible cases, for 51 couples and 15 single parents. We failed to determine which allele was transmitted in three cases for *IL4* and in four cases for *IL5* because of the heterozygosity of single parents and their children.

For the G/C 3′-UTR polymorphism of the *IL4* gene, 48 cases of alleles transmitted from heterozygous parents to their affected children were revealed: 21 for allele G and 27 for allele C (Table 1). The deviation from equal segregation was non-significant (TDT $= 0.750$, $p = 0.476 \pm 0.0041$). For the C-703T polymorphism of the *IL5* gene, 52 cases of alleles transmitted from heterozygous parents to their affected children were revealed: 34 for allele C and 18 for allele T (Table 1). This distribution deviated significantly from equal segregation (TDT $= 4.923$, $p = 0.007 \pm 0.0007$).

These data suggest a significant association of the C-703 allele of the *IL5* gene with atopic BA in Russians. Since the C-703T transition is located in the promoter region of *IL5* it could affect the expression of the gene and, hence, influence the level of interleukin-5. The important role of this cytokine in BA pathogenesis was determined by its ability to activate eosinophils, which in turn participate in the development and prolongation of airway inflammation (Chung and Barnes, 1999). Taking into account that the association with BA was found for the C-703 allele, it could be assumed that this variant of the gene was expressed at a higher level in comparison with that of the T-703 allele and caused an increased production of interleukin-5, predisposing to BA.

There was no prior evidence for an association of *IL5* common polymorphisms with BA. The analyses of sequences of *IL5* and *IL5RA* (encoding an $\alpha$-chain of the interleukin-5 receptor) genes in a sample of 30 patients with BA and 30 healthy individuals did not reveal any variants, and it was concluded that polymorphisms of these genes were unlikely to contribute to the BA phenotype (Pereira *et al.*, 1998). Analysis of the association of the transition C-703T in *IL5* with familial eosinophilia also yielded negative results (Rioux *et al.*, 1998). Thus, the association of C-703T with asthma in Russians provides the first evidence for a possible contribution of common polymorphism in the *IL5* gene with a complex pathological

**Table 1.** Distribution of alleles of the *IL4* and *IL5* genes, transmitted to probands with atopic bronchial asthma from their heterozygous parents

| | | Number of alleles, transmitted to affected offspring from heterozygous parents | | | |
|---|---|---|---|---|---|
| Gene | Polymorphism | Observed | Expected | TDT* | $p$** |
| *IL4* | G/C 3′-UTR | G: 21 | 24 | 0.750 | 0.474 ± 0.0041 |
| | | C: 27 | 24 | | |
| *IL5* | C-703T | C: 34 | 26 | 4.923 | 0.007 ± 0.0007 |
| | | T: 18 | 26 | | |

* Transmission/disequilibrium test statistics (Spielman *et al.*, 1993).
** Significance level for TDT (Monte Carlo-based approach; 15 000 simulations were used).

Copyright © 2003 John Wiley & Sons, Ltd. 

condition, the manifestation of which strongly depends on eosinophil expansion.

In addition to the analysis of the association of the polymorphisms in the *IL4* and *IL5* genes with BA, their association with the severity of the disease was also evaluated. For this purpose, 55 unrelated patients with mild and severe BA were randomly chosen from the family sample. In addition, 34 unrelated patients with the severe form of the disease were included. The sample analysed contained 40 patients with mild BA and 49 with severe BA.

By comparison of genotype frequencies, a significant negative association of genotypes GG *IL4* (OR = 0.39, 95% CI = 0.15–0.99, $p = 0.035$) and TT *IL5* (OR = 0.00; 95% CI = 0.00–1.09, $p = 0.031$) with mild asthma was found (Table 2). Furthermore, a trend to a positive association of the genotype GC *IL4* with mild BA (OR = 2.52, 95% CI = 0.98–6.57, $p = 0.052$) and a non-significant prevalence of the allele G *IL4* in patients with severe forms of the disease in comparison with that in individuals with mild asthma (OR = 0.53, 95% CI = 0.26–1.11, $p = 0.085$) were observed.

These data suggest that genotype GG *IL4* is a risk factor for the severe course of atopic BA. Taking into account the association of allele C-703 of *IL5* with BA, the significant prevalence of the genotype TT *IL5* in patients with severe asthma in comparison to those with mild forms of the disorder appears quite paradoxical: it is difficult to understand why one allele would contribute to the disease, whereas the alternative allele would provoke its severe course. In the samples investigated, four individuals with the genotype GG *IL4* had also the genotype TT *IL5* (about 67%). Hence, it is conceivable that the observed association of the genotype TT *IL5* with severe BA is accidental.

Interleukin-4 is the key cytokine in allergic responses. It induces differentiation of $CD4^+$ T-lymphocytes to Th2-helper cells, and accounts for immunoglobulin heavy chain $\mu$ to $\varepsilon$ isotype switching, thus resulting in IgE synthesis (Paul and Seder, 1994; Stavnezer, 1996). An amplification of interleukin-4 production is the main stimulus for the increase and prolongation of IgE synthesis in patients with atopic diseases (Chung and Barnes, 1999). Probably, interleukin-4 hyperproduction also can cause the severe course of BA. The transversion of nucleotide G to C that we have studied is located outside of the coding region of the *IL4* gene, therefore it could influence the gene expression profile. The nearest control elements to the G/C polymorphism in the *IL4* gene are the transcriptional stop-codon TAA (nucleotides 8676–8678) and the polyA signal AATAAA (nucleotides 9639–9644), but it is not known to date whether the G/C polymorphism we have studied influences the functioning of these elements.

The results presented may be of interest for prediction on the prognosis of disease manifestation. If a genotype is expected to contribute to a specific clinical phenotype, one might be able to predict the probability of its realization and to choose the

**Table 2.** Number and percentage (in parentheses) of genotypes and allelic frequencies (±SE) of the *IL4* and *IL5* gene polymorphisms in patients with different severities of atopic bronchial asthma (BA)

| | | | Severity of BA | | | |
|---|---|---|---|---|---|---|
| **Gene** | **Polymorphism** | | **Mild** | **Severe** | **OR (95% CI)*** | ***p**** |
| *IL4* | G/C 3′-UTR | GG | 16 (40.0) | 31 (63.3) | 0.39 (0.15–0.99) | 0.035 |
| | | GC | 22 (55.0) | 16 (32.6) | 2.52 (0.98–6.57) | 0.052 |
| | | CC | 2 (5.0) | 2 (4.1) | 1.24 (0.12–13.06) | 0.999 |
| | | G | 0.675 ± 0.052 | 0.796 ± 0.410 | 0.53 (0.26–1.11) | 0.085 |
| *IL5* | C-703T | CC | 25 (62.5) | 26 (53.1) | 1.47 (0.58–3.78) | 0.397 |
| | | CT | 15 (37.5) | 17 (34.7) | 1.13 (0.43–2.95) | 0.827 |
| | | TT | 0 (0.00) | 6 (12.2) | 0.00 (0.00–1.09) | 0.031 |
| | | C | 0.813 ± 0.044 | 0.704 ± 0.046 | 1.82 (0.85–3.94) | 0.117 |

* Odds ratio with 95% confidential interval (in parentheses) of mild BA in comparison with the severe form of the disorder in probands with designated genotype or allele.
** Significance level by two-tailed Fisher's exact test.

Copyright © 2003 John Wiley & Sons, Ltd. 

appropriate therapy. To check the prognostic value of the G/C 3′-UTR polymorphism in the *IL4* gene in relation to BA severity, a 'genotype' diagnosis was stated for unrelated individuals: the severe form of the disease was applied to men with the GG genotype, and mild BA form was applied to those with the GC genotype. Four homozygotes for the allele C *IL4* were excluded as non-informative, so 85 subjects in total were included in the sample. In this group, an estimate of correspondence between 'genotype' classification and clinical diagnosis was carried out. The analysis of the contingency table revealed a successful conformity of the diagnostic approaches: McNemar's $X^2$ with continuity correction constituted 0.03, d.f. $= 1, p = 0.859$ (Table 3). Coincidence of diagnoses was obtained in 53 cases out of 85 (62.4%). Thus, we wish to propose that the G/C 3′-UTR polymorphism in the *IL4* gene might serve a prognostic purpose for BA severity. However, it can be used only as an additional predictive criterion, because its use as a marker of severe or mild BA course failed in 37.6% of cases.

In the present study, two common polymorphisms in the regulatory regions of the *IL4* and *IL5* genes were analysed. These genes have been reported as the candidate genes of atopic BA, and their protein products are the key molecules for such pathogenetic components of the disease as allergy and eosinophilic inflammation. The C-703T polymorphism in the *IL5* gene is associated with BA (allele C) and the G/C 3′-UTR polymorphism in the *IL4* gene is associated with the severe form of the disease. To date, there are no functional data on these polymorphisms, therefore the mechanisms of their association with BA are presently unknown. To clarify these mechanisms it will be necessary to design transgenic cell lines to estimate the expression levels of the genes depending on their genotypes at these polymorphic sites.

**Table 3.** Distribution of individuals with different severity of bronchial asthma by clinical and 'genotype' diagnosis*

| | Clinical diagnosis | |
|---|---|---|
| **'Genotype' diagnosis** | **Severe asthma** | **Mild asthma** |
| Severe asthma | 31 | 16 |
| Mild asthma | 16 | 22 |

* The 'genotype' diagnosis was stated by G/C 3′-UTR *IL4* polymorphism: the severe form of the disease was applied to individuals with genotype GG *IL4*, and the mild form was applied to those with genotype GC *IL4*. Homozygotes on allele C *IL4* were excluded as non-informative. Differences between clinical and 'genotype' diagnoses are non-significant: McNemar's $X^2$ with continuity correction constituted 0.03, d.f. $= 1$, $p = 0.859$.

### Acknowledgements

The authors thank Professor J. M. Hopkin and Dr T. Shirakawa of the Experimental Medicine Unit of the University of Wales Swansea (UK) for their kind provision of the techniques for genotyping the *IL4* and *IL5* polymorphisms. This work was partially supported by the Russian Foundation for Basic Research Grants 01-04-48213 and 00-15-97876.

### References

Chung KF, Barnes PJ. 1999. Cytokines in asthma. *Thorax* **54**: 825–857.

Cookson WOCM, Moffatt MF. 2000. Genetics of asthma and allergic disease. *Hum Mol Genet* **9**: 2359–2364.

National Heart, Lung and Blood Institute. 1995. Global initiative for asthma. Global strategy for asthma management and prevention. NHLI/WHO Workshop report.

Paul WE, Seder RA. 1994. Lymphocyte response and cytokines. *Cell* **76**: 241–251.

Pereira E, Goldblatt J, Rye P, *et al*. 1998. Mutation analysis of interleukin-5 in asthmatic cohort. *Hum Mutat* **11**: 51–54.

Rioux JD, Stone VA, Daly M, *et al*. 1998. Familial eosinophilia maps to the cytokine gene cluster on human chromosomal region 5q31–q33. *Am J Hum Genet* **63**: 1086–1094.

Spielman RS, McGinnis RE, Ewens WJ. 1993. Transmission test for linkage disequilibrium: the insulin gene region and insulin-dependent diabetes mellitus (IDDM). *Am J Hum Genet* **52**: 506–516.

Stavnezer J. 1996. Antibody class switching. *Adv Immunol* **61**: 79–146.

Copyright © 2003 John Wiley & Sons, Ltd.
